# GEAR 2.0 ‐ Detection of Dementia and Cognitive Impairment

**DOI:** 10.1002/alz.091835

**Published:** 2025-01-09

**Authors:** Ula Hwang

**Affiliations:** ^1^ NYU Langone Health, New York, NY, USA; James J. Peter’s VA Medical Center, Bronx, NY USA

## Abstract

Older adults use the emergency department (ED) as an important source of acute medical care, making 20+ million visits annually. Persons living with dementia are twice as likely to use the ED and 1.5 times more likely to have an avoidable visit. When in the ED, they often struggle with the fast‐paced setting and may not be able to give a complete medical history. These challenges, along with other adverse events, put patients with dementia at greater risk for poor outcomes. Yet emergency care for older adults is suboptimal, and care is especially poor for older adults with dementia, even though these adults seek ED‐based care more regularly than matched controls.

Three decades of research has shown that dementia is under‐recognized in emergency departments, despite a proliferation of screening tools. Under‐recognition of dementia in the ED leads to under‐recognition in inpatient services, which has broad‐reaching consequences, such as longer hospital stays, lower patient satisfaction, accelerated cognitive declines, and increased health care costs. Better detection of dementia may improve the ED staff’s ability to implement interventions that can reduce the rate of cognitive decline and improve care coordination and patient safety.

This session will review the literature of current and past dementia detection approaches and discuss top research questions generated by the patients, care partners and members of the transdisciplinary GEAR Detection Work Group.

